# (Biphenyl-2-yl)bromidobis(2-methyl­tetra­hydro­furan-κ*O*)magnesium(II)

**DOI:** 10.1107/S1600536809012185

**Published:** 2009-04-08

**Authors:** Simon Nordschild, D. Wohlgemuth, Michael Bolte

**Affiliations:** a Chemetall GmbH, Lithium Division, Trakehner Strasse 3, 60487 Frankfurt am Main, Germany; b Institut für Anorganische Chemie, J. W. Goethe-Universität Frankfurt, Max-von-Laue-Strasse 7, 60438 Frankfurt/Main, Germany

## Abstract

In the title Grignard reagent, [MgBr(C_12_H_9_)(C_5_H_10_O)_2_], the Mg centre adopts a distorted tetra­hedral MgCO_2_Br arrangement. The dihedral angle between the two aromatic rings of the biphenyl residue is 44.00 (14)°. Each mol­ecule incorporates one *R*- and one *S*-configured 2-methyl­tetra­hydro­furan mol­ecule.

## Related literature

For background to Grignard-type compounds, see Elschenbroich (2008 ▶); Schwetlick (1996 ▶); Silverman & Rakita (1996 ▶).
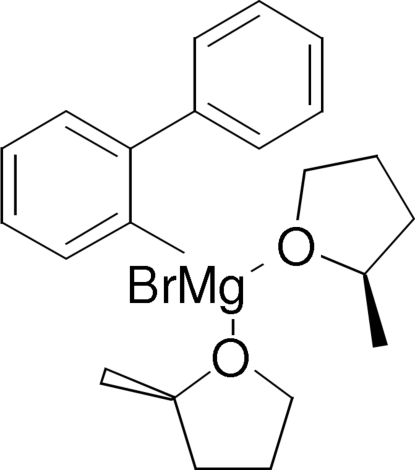


## Experimental

### 

#### Crystal data

[MgBr(C_12_H_9_)(C_5_H_10_O)_2_]
*M* *_r_* = 429.67
Monoclinic, 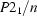

*a* = 11.6887 (5) Å
*b* = 16.8061 (9) Å
*c* = 11.7888 (5) Å
β = 103.757 (4)°
*V* = 2249.38 (18) Å^3^
*Z* = 4
Mo *K*α radiation
μ = 1.87 mm^−1^
*T* = 173 K
0.29 × 0.28 × 0.26 mm

#### Data collection

Stoe IPDSII two-circle diffractometer
Absorption correction: multi-scan (*MULABS*; Spek, 2003 ▶; Blessing, 1995 ▶) *T*_min_ = 0.614, *T*_max_ = 0.642
54470 measured reflections
4107 independent reflections
3484 reflections with *I* > 2σ(*I*)
*R*_int_ = 0.069

#### Refinement

*R*[*F* ^2^ > 2σ(*F* ^2^)] = 0.045
*wR*(*F* ^2^) = 0.108
*S* = 1.05
4107 reflections
236 parameters
H-atom parameters constrained
Δρ_max_ = 0.76 e Å^−3^
Δρ_min_ = −0.41 e Å^−3^

### 

Data collection: *X-AREA* (Stoe & Cie, 2001 ▶); cell refinement: *X-AREA*; data reduction: *X-AREA*; program(s) used to solve structure: *SHELXS97* (Sheldrick, 2008 ▶); program(s) used to refine structure: *SHELXL97* (Sheldrick, 2008 ▶); molecular graphics: *XP* in *SHELXTL-Plus* (Sheldrick, 2008 ▶); software used to prepare material for publication: *SHELXL97*.

## Supplementary Material

Crystal structure: contains datablocks I, global. DOI: 10.1107/S1600536809012185/hb2933sup1.cif

Structure factors: contains datablocks I. DOI: 10.1107/S1600536809012185/hb2933Isup2.hkl

Additional supplementary materials:  crystallographic information; 3D view; checkCIF report

## Figures and Tables

**Table:** 

Mg1—O1	2.022 (2)
Mg1—O11	2.030 (2)
Mg1—C21	2.143 (3)
Mg1—Br1	2.4750 (10)

**Table:** 

O1—Mg1—O11	97.02 (10)
O1—Mg1—C21	114.41 (11)
O11—Mg1—C21	106.87 (11)
O1—Mg1—Br1	109.97 (9)
O11—Mg1—Br1	104.75 (7)
C21—Mg1—Br1	120.53 (8)

**Fig. 1.**
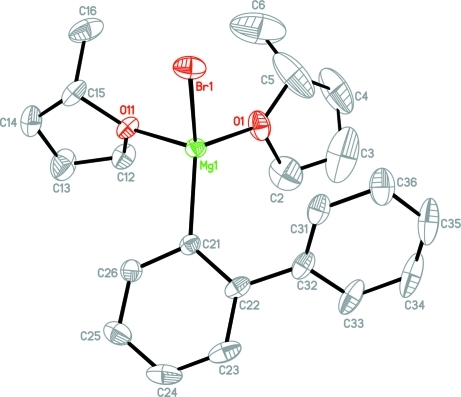
Perspective view of (I) with displacement ellipsoids drawn at the 50% probability level; H atoms omitted for clarity.

## supplementary crystallographic information

### Comment

The title compound is a Grignard reagent, a kind of compounds which is widely used for C—C bond formation or as base in organic chemistry. The Mg centre is four coordinate in a distorted tetrahedral fashion. The bond angles range from 97.02 (1)° for O—Mg—O to 120.53 (8)° for C—Mg—Br. The bond lengths are 2.4750 (10)Å for Mg—Br, 2.143 (3)Å for Mg—C and 2.022 (2)° and 2.030 (2)Å for the Mg—O bonds. The dihedral angles between the two aromatic rings of the biphenyl residue is 44.00 (14)°.

### Experimental

This compound is commercially available from Chemetall GmbH (CAS 82214–69-5, Product No. 408562).  Colourless blocks of (I) were obtained from a solution due to long term storage at ambient temperature.

### Refinement

The H atoms bonded were geometrically positioned and refined with fixed individual displacement parameters [U(H) = 1.2 *U*_eq_(C) or U(H) = 1.5 *U*_eq_(C_methyl_)] using a riding model with C_aromatic_—H = 0.95 Å, C_methyl_—H = 0.98 Å, C_methylene_—H = 0.99Å and C_tertiary_—H = 1.00 Å.

### Crystal data

**Table:**

[MgBr(C_12_H_9_)(C_5_H_10_O)_2_]	*F*(000) = 896
*M**_r_* = 429.67	*D*_x_ = 1.269 Mg m^−^^3^
Monoclinic, *P*2_1_/*n*	Mo *K*α radiation, λ = 0.71073 Å
Hall symbol: -P 2yn	Cell parameters from 40381 reflections
*a* = 11.6887 (5) Å	θ = 3.6–26.6°
*b* = 16.8061 (9) Å	µ = 1.87 mm^−^^1^
*c* = 11.7888 (5) Å	*T* = 173 K
β = 103.757 (4)°	Block, colourless
*V* = 2249.38 (18) Å^3^	0.29 × 0.28 × 0.26 mm
*Z* = 4	

### Data collection

**Table:**

Stoe IPDSII two-circle diffractometer	4107 independent reflections
Radiation source: fine-focus sealed tube	3484 reflections with *I* > 2σ(*I*)
graphite	*R*_int_ = 0.069
ω scans	θ_max_ = 25.4°, θ_min_ = 3.6°
Absorption correction: multi-scan (*MULABS*; Spek, 2003; Blessing, 1995)	*h* = −14→14
*T*_min_ = 0.614, *T*_max_ = 0.642	*k* = −20→20
54470 measured reflections	*l* = −14→14

### Refinement

**Table:**

Refinement on *F*^2^	Secondary atom site location: difference Fourier map
Least-squares matrix: full	Hydrogen site location: inferred from neighbouring sites
*R*[*F*^2^ > 2σ(*F*^2^)] = 0.045	H-atom parameters constrained
*wR*(*F*^2^) = 0.108	*w* = 1/[σ^2^(*F*_o_^2^) + (0.0462*P*)^2^ + 2.3133*P*] where *P* = (*F*_o_^2^ + 2*F*_c_^2^)/3
*S* = 1.05	(Δ/σ)_max_ < 0.001
4107 reflections	Δρ_max_ = 0.76 e Å^−^^3^
236 parameters	Δρ_min_ = −0.41 e Å^−^^3^
0 restraints	Extinction correction: *SHELXL97* (Sheldrick, 2008), Fc^*^=kFc[1+0.001xFc^2^λ^3^/sin(2θ)]^-1/4^
Primary atom site location: structure-invariant direct methods	Extinction coefficient: 0.0043 (6)

### Special details

**Table:**

Geometry. All e.s.d.'s (except the e.s.d. in the dihedral angle between two l.s. planes) are estimated using the full covariance matrix. The cell e.s.d.'s are taken into account individually in the estimation of e.s.d.'s in distances, angles and torsion angles; correlations between e.s.d.'s in cell parameters are only used when they are defined by crystal symmetry. An approximate (isotropic) treatment of cell e.s.d.'s is used for estimating e.s.d.'s involving l.s. planes.
Refinement. Refinement of *F*^2^ against ALL reflections. The weighted *R*-factor *wR* and goodness of fit *S* are based on *F*^2^, conventional *R*-factors *R* are based on *F*, with *F* set to zero for negative *F*^2^. The threshold expression of *F*^2^ > σ(*F*^2^) is used only for calculating *R*-factors(gt) *etc*. and is not relevant to the choice of reflections for refinement. *R*-factors based on *F*^2^ are statistically about twice as large as those based on *F*, and *R*- factors based on ALL data will be even larger.

### Fractional atomic coordinates and isotropic or equivalent isotropic displacement parameters (Å^2^)

**Table:**

	*x*	*y*	*z*	*U*_iso_*/*U*_eq_	
Mg1	0.56815 (8)	0.31486 (6)	0.70780 (8)	0.0349 (2)	
Br1	0.60093 (3)	0.24360 (2)	0.89652 (3)	0.05398 (15)	
O1	0.4214 (2)	0.38260 (15)	0.6841 (2)	0.0572 (6)	
C2	0.4112 (4)	0.4578 (4)	0.6045 (5)	0.0967 (17)	
H2A	0.4898	0.4781	0.6011	0.116*	
H2B	0.3649	0.4465	0.5243	0.116*	
C3	0.3515 (7)	0.5122 (4)	0.6625 (8)	0.159 (4)	
H3A	0.3114	0.5542	0.6085	0.190*	
H3B	0.4067	0.5376	0.7296	0.190*	
C4	0.2618 (6)	0.4595 (5)	0.7036 (7)	0.145 (3)	
H4A	0.2312	0.4860	0.7653	0.173*	
H4B	0.1951	0.4450	0.6379	0.173*	
C5	0.3367 (5)	0.3866 (6)	0.7512 (5)	0.143 (3)	
H5	0.3763	0.3948	0.8354	0.172*	
C6	0.2702 (6)	0.3147 (6)	0.7372 (7)	0.170 (4)	
H6A	0.3212	0.2705	0.7720	0.256*	
H6B	0.2383	0.3044	0.6539	0.256*	
H6C	0.2052	0.3199	0.7762	0.256*	
O11	0.50159 (19)	0.23129 (11)	0.58553 (18)	0.0412 (5)	
C12	0.4587 (3)	0.2531 (2)	0.4632 (3)	0.0536 (8)	
H12A	0.4940	0.3040	0.4464	0.064*	
H12B	0.3718	0.2587	0.4430	0.064*	
C13	0.4955 (5)	0.1870 (3)	0.3968 (4)	0.0852 (15)	
H13A	0.4355	0.1777	0.3231	0.102*	
H13B	0.5713	0.1998	0.3774	0.102*	
C14	0.5080 (4)	0.1158 (2)	0.4719 (4)	0.0672 (11)	
H14A	0.5833	0.0884	0.4732	0.081*	
H14B	0.4426	0.0782	0.4422	0.081*	
C15	0.5056 (3)	0.14444 (18)	0.5944 (3)	0.0521 (9)	
H15	0.5799	0.1279	0.6508	0.063*	
C16	0.4023 (4)	0.1148 (3)	0.6359 (4)	0.0790 (13)	
H16A	0.4065	0.1351	0.7147	0.119*	
H16B	0.4032	0.0565	0.6373	0.119*	
H16C	0.3294	0.1334	0.5829	0.119*	
C21	0.7121 (2)	0.37048 (16)	0.6550 (2)	0.0341 (6)	
C22	0.7636 (2)	0.44648 (17)	0.6866 (3)	0.0395 (7)	
C23	0.8611 (3)	0.4719 (2)	0.6460 (3)	0.0549 (9)	
H23	0.8928	0.5234	0.6666	0.066*	
C24	0.9121 (3)	0.4234 (3)	0.5767 (4)	0.0627 (11)	
H24	0.9794	0.4411	0.5518	0.075*	
C25	0.8648 (3)	0.3495 (2)	0.5439 (3)	0.0542 (9)	
H25	0.8986	0.3159	0.4959	0.065*	
C26	0.7668 (3)	0.32490 (18)	0.5821 (3)	0.0419 (7)	
H26	0.7345	0.2741	0.5577	0.050*	
C31	0.6804 (3)	0.4711 (2)	0.8611 (3)	0.0483 (8)	
H31	0.6914	0.4163	0.8802	0.058*	
C32	0.7136 (3)	0.50025 (17)	0.7624 (3)	0.0442 (8)	
C33	0.6971 (4)	0.5819 (2)	0.7384 (3)	0.0638 (11)	
H33	0.7205	0.6039	0.6732	0.077*	
C34	0.6474 (5)	0.6307 (2)	0.8081 (4)	0.0854 (16)	
H34	0.6354	0.6855	0.7890	0.102*	
C35	0.6154 (4)	0.6014 (3)	0.9041 (4)	0.0853 (16)	
H35	0.5821	0.6356	0.9517	0.102*	
C36	0.6318 (4)	0.5206 (2)	0.9319 (3)	0.0656 (11)	
H36	0.6099	0.4997	0.9986	0.079*	

### Atomic displacement parameters (Å^2^)

**Table:**

	*U*^11^	*U*^22^	*U*^33^	*U*^12^	*U*^13^	*U*^23^
Mg1	0.0308 (5)	0.0364 (5)	0.0377 (5)	−0.0051 (4)	0.0085 (4)	−0.0023 (4)
Br1	0.0496 (2)	0.0716 (3)	0.0401 (2)	−0.01271 (17)	0.00923 (14)	0.00894 (16)
O1	0.0412 (13)	0.0673 (16)	0.0586 (15)	0.0132 (11)	0.0029 (11)	−0.0154 (12)
C2	0.059 (3)	0.116 (4)	0.106 (4)	0.027 (3)	0.002 (3)	0.014 (3)
C3	0.129 (6)	0.114 (5)	0.174 (8)	0.051 (5)	−0.082 (6)	−0.039 (5)
C4	0.103 (5)	0.204 (8)	0.133 (6)	0.102 (6)	0.039 (4)	−0.001 (5)
C5	0.080 (4)	0.269 (10)	0.082 (4)	0.081 (5)	0.023 (3)	−0.001 (5)
C6	0.086 (5)	0.277 (12)	0.145 (7)	−0.072 (6)	0.022 (4)	0.027 (7)
O11	0.0477 (12)	0.0308 (11)	0.0408 (11)	−0.0064 (9)	0.0017 (9)	0.0021 (8)
C12	0.065 (2)	0.0477 (19)	0.0407 (17)	−0.0002 (17)	−0.0019 (15)	0.0043 (15)
C13	0.137 (5)	0.065 (3)	0.054 (2)	−0.002 (3)	0.024 (3)	−0.013 (2)
C14	0.072 (3)	0.050 (2)	0.075 (3)	0.0072 (19)	0.009 (2)	−0.0172 (19)
C15	0.054 (2)	0.0317 (16)	0.061 (2)	−0.0068 (14)	−0.0061 (16)	0.0057 (15)
C16	0.090 (3)	0.069 (3)	0.073 (3)	−0.043 (2)	0.010 (2)	0.003 (2)
C21	0.0299 (13)	0.0292 (14)	0.0409 (15)	0.0013 (11)	0.0038 (11)	0.0036 (12)
C22	0.0312 (14)	0.0343 (15)	0.0451 (17)	−0.0061 (12)	−0.0063 (12)	0.0082 (13)
C23	0.0436 (19)	0.054 (2)	0.058 (2)	−0.0204 (16)	−0.0055 (16)	0.0150 (17)
C24	0.0345 (17)	0.086 (3)	0.067 (2)	−0.0100 (18)	0.0113 (16)	0.025 (2)
C25	0.0435 (18)	0.066 (2)	0.057 (2)	0.0104 (17)	0.0198 (16)	0.0174 (18)
C26	0.0397 (16)	0.0364 (16)	0.0507 (18)	0.0063 (13)	0.0129 (14)	0.0064 (13)
C31	0.0481 (18)	0.0392 (17)	0.0478 (19)	0.0046 (14)	−0.0079 (14)	−0.0072 (14)
C32	0.0417 (16)	0.0341 (16)	0.0450 (18)	−0.0045 (13)	−0.0132 (13)	−0.0052 (13)
C33	0.082 (3)	0.0333 (18)	0.055 (2)	−0.0038 (17)	−0.0242 (19)	−0.0040 (16)
C34	0.124 (4)	0.038 (2)	0.065 (3)	0.015 (2)	−0.035 (3)	−0.017 (2)
C35	0.103 (4)	0.064 (3)	0.066 (3)	0.033 (2)	−0.025 (3)	−0.034 (2)
C36	0.069 (2)	0.068 (2)	0.049 (2)	0.013 (2)	−0.0076 (18)	−0.0172 (18)

### Geometric parameters (Å, °)

**Table:**

Mg1—O1	2.022 (2)	C14—H14A	0.9900
Mg1—O11	2.030 (2)	C14—H14B	0.9900
Mg1—C21	2.143 (3)	C15—C16	1.492 (5)
Mg1—Br1	2.4750 (10)	C15—H15	1.0000
O1—C5	1.408 (6)	C16—H16A	0.9800
O1—C2	1.562 (6)	C16—H16B	0.9800
C2—C3	1.421 (9)	C16—H16C	0.9800
C2—H2A	0.9900	C21—C26	1.412 (4)
C2—H2B	0.9900	C21—C22	1.424 (4)
C3—C4	1.536 (11)	C22—C23	1.403 (5)
C3—H3A	0.9900	C22—C32	1.485 (5)
C3—H3B	0.9900	C23—C24	1.385 (6)
C4—C5	1.533 (9)	C23—H23	0.9500
C4—H4A	0.9900	C24—C25	1.377 (6)
C4—H4B	0.9900	C24—H24	0.9500
C5—C6	1.425 (11)	C25—C26	1.390 (4)
C5—H5	1.0000	C25—H25	0.9500
C6—H6A	0.9800	C26—H26	0.9500
C6—H6B	0.9800	C31—C36	1.393 (5)
C6—H6C	0.9800	C31—C32	1.399 (5)
O11—C12	1.456 (4)	C31—H31	0.9500
O11—C15	1.463 (4)	C32—C33	1.404 (4)
C12—C13	1.481 (5)	C33—C34	1.384 (6)
C12—H12A	0.9900	C33—H33	0.9500
C12—H12B	0.9900	C34—C35	1.366 (7)
C13—C14	1.475 (6)	C34—H34	0.9500
C13—H13A	0.9900	C35—C36	1.398 (6)
C13—H13B	0.9900	C35—H35	0.9500
C14—C15	1.529 (5)	C36—H36	0.9500
			
O1—Mg1—O11	97.02 (10)	C13—C14—C15	106.8 (3)
O1—Mg1—C21	114.41 (11)	C13—C14—H14A	110.4
O11—Mg1—C21	106.87 (11)	C15—C14—H14A	110.4
O1—Mg1—Br1	109.97 (9)	C13—C14—H14B	110.4
O11—Mg1—Br1	104.75 (7)	C15—C14—H14B	110.4
C21—Mg1—Br1	120.53 (8)	H14A—C14—H14B	108.6
C5—O1—C2	109.6 (4)	O11—C15—C16	110.0 (3)
C5—O1—Mg1	129.5 (3)	O11—C15—C14	104.7 (3)
C2—O1—Mg1	118.7 (2)	C16—C15—C14	113.8 (3)
C3—C2—O1	102.0 (5)	O11—C15—H15	109.4
C3—C2—H2A	111.4	C16—C15—H15	109.4
O1—C2—H2A	111.4	C14—C15—H15	109.4
C3—C2—H2B	111.4	C15—C16—H16A	109.5
O1—C2—H2B	111.4	C15—C16—H16B	109.5
H2A—C2—H2B	109.2	H16A—C16—H16B	109.5
C2—C3—C4	103.3 (6)	C15—C16—H16C	109.5
C2—C3—H3A	111.1	H16A—C16—H16C	109.5
C4—C3—H3A	111.1	H16B—C16—H16C	109.5
C2—C3—H3B	111.1	C26—C21—C22	114.9 (3)
C4—C3—H3B	111.1	C26—C21—Mg1	116.1 (2)
H3A—C3—H3B	109.1	C22—C21—Mg1	128.9 (2)
C5—C4—C3	101.7 (5)	C23—C22—C21	120.8 (3)
C5—C4—H4A	111.4	C23—C22—C32	119.2 (3)
C3—C4—H4A	111.4	C21—C22—C32	120.0 (3)
C5—C4—H4B	111.4	C24—C23—C22	121.3 (3)
C3—C4—H4B	111.4	C24—C23—H23	119.3
H4A—C4—H4B	109.3	C22—C23—H23	119.3
O1—C5—C6	109.4 (6)	C25—C24—C23	119.7 (3)
O1—C5—C4	104.4 (6)	C25—C24—H24	120.1
C6—C5—C4	112.8 (7)	C23—C24—H24	120.1
O1—C5—H5	110.0	C24—C25—C26	119.0 (3)
C6—C5—H5	110.0	C24—C25—H25	120.5
C4—C5—H5	110.0	C26—C25—H25	120.5
C5—C6—H6A	109.5	C25—C26—C21	124.2 (3)
C5—C6—H6B	109.5	C25—C26—H26	117.9
H6A—C6—H6B	109.5	C21—C26—H26	117.9
C5—C6—H6C	109.5	C36—C31—C32	121.6 (3)
H6A—C6—H6C	109.5	C36—C31—H31	119.2
H6B—C6—H6C	109.5	C32—C31—H31	119.2
C12—O11—C15	108.6 (2)	C31—C32—C33	117.2 (3)
C12—O11—Mg1	120.80 (18)	C31—C32—C22	121.0 (3)
C15—O11—Mg1	129.73 (19)	C33—C32—C22	121.8 (3)
O11—C12—C13	105.0 (3)	C34—C33—C32	121.1 (4)
O11—C12—H12A	110.7	C34—C33—H33	119.5
C13—C12—H12A	110.7	C32—C33—H33	119.5
O11—C12—H12B	110.7	C35—C34—C33	121.0 (4)
C13—C12—H12B	110.7	C35—C34—H34	119.5
H12A—C12—H12B	108.8	C33—C34—H34	119.5
C14—C13—C12	106.9 (3)	C34—C35—C36	119.7 (4)
C14—C13—H13A	110.3	C34—C35—H35	120.1
C12—C13—H13A	110.3	C36—C35—H35	120.1
C14—C13—H13B	110.3	C31—C36—C35	119.4 (4)
C12—C13—H13B	110.3	C31—C36—H36	120.3
H13A—C13—H13B	108.6	C35—C36—H36	120.3
			
O11—Mg1—O1—C5	101.5 (5)	C13—C14—C15—C16	115.6 (4)
C21—Mg1—O1—C5	−146.4 (5)	O1—Mg1—C21—C26	−130.5 (2)
Br1—Mg1—O1—C5	−7.1 (5)	O11—Mg1—C21—C26	−24.4 (2)
O11—Mg1—O1—C2	−97.5 (3)	Br1—Mg1—C21—C26	94.8 (2)
C21—Mg1—O1—C2	14.7 (3)	O1—Mg1—C21—C22	52.0 (3)
Br1—Mg1—O1—C2	154.0 (3)	O11—Mg1—C21—C22	158.2 (2)
C5—O1—C2—C3	20.6 (6)	Br1—Mg1—C21—C22	−82.6 (3)
Mg1—O1—C2—C3	−144.0 (4)	C26—C21—C22—C23	0.3 (4)
O1—C2—C3—C4	−38.6 (5)	Mg1—C21—C22—C23	177.7 (2)
C2—C3—C4—C5	43.7 (7)	C26—C21—C22—C32	179.6 (3)
C2—O1—C5—C6	127.7 (6)	Mg1—C21—C22—C32	−2.9 (4)
Mg1—O1—C5—C6	−69.9 (7)	C21—C22—C23—C24	−1.6 (5)
C2—O1—C5—C4	6.8 (7)	C32—C22—C23—C24	179.0 (3)
Mg1—O1—C5—C4	169.2 (4)	C22—C23—C24—C25	1.7 (5)
C3—C4—C5—O1	−29.7 (8)	C23—C24—C25—C26	−0.4 (5)
C3—C4—C5—C6	−148.4 (7)	C24—C25—C26—C21	−1.0 (5)
O1—Mg1—O11—C12	60.3 (3)	C22—C21—C26—C25	1.1 (4)
C21—Mg1—O11—C12	−57.9 (3)	Mg1—C21—C26—C25	−176.7 (2)
Br1—Mg1—O11—C12	173.1 (2)	C36—C31—C32—C33	0.8 (5)
O1—Mg1—O11—C15	−131.4 (3)	C36—C31—C32—C22	−179.0 (3)
C21—Mg1—O11—C15	110.4 (3)	C23—C22—C32—C31	−136.9 (3)
Br1—Mg1—O11—C15	−18.6 (3)	C21—C22—C32—C31	43.7 (4)
C15—O11—C12—C13	−28.8 (4)	C23—C22—C32—C33	43.3 (4)
Mg1—O11—C12—C13	141.7 (3)	C21—C22—C32—C33	−136.1 (3)
O11—C12—C13—C14	25.2 (5)	C31—C32—C33—C34	−1.6 (5)
C12—C13—C14—C15	−12.7 (5)	C22—C32—C33—C34	178.2 (3)
C12—O11—C15—C16	−102.0 (3)	C32—C33—C34—C35	1.6 (6)
Mg1—O11—C15—C16	88.6 (3)	C33—C34—C35—C36	−0.7 (7)
C12—O11—C15—C14	20.7 (4)	C32—C31—C36—C35	0.1 (5)
Mg1—O11—C15—C14	−148.7 (2)	C34—C35—C36—C31	−0.1 (6)
C13—C14—C15—O11	−4.6 (4)		
